# Privately computing set-maximal matches in genomic data

**DOI:** 10.1186/s12920-020-0718-x

**Published:** 2020-07-21

**Authors:** Katerina Sotiraki, Esha Ghosh, Hao Chen

**Affiliations:** 1 MIT, CSAIL, 32 Vassar street, Cambridge, 02139 MA USA; 2 Microsoft Research, 14820 NE 36th Street, Building 99, Redmond, 98052 WA USA

**Keywords:** Set-maximal match, Privacy, Secret sharing

## Abstract

**Background:**

Finding long matches in deoxyribonucleic acid (DNA) sequences in large aligned genetic sequences is a problem of great interest. A paradigmatic application is the identification of distant relatives via large common subsequences in DNA data. However, because of the sensitive nature of genomic data such computations without security consideration might compromise the privacy of the individuals involved.

**Methods:**

The secret sharing technique enables the computation of matches while respecting the privacy of the inputs of the parties involved. This method requires interaction that depends on the circuit depth needed for the computation.

**Results:**

We design a new depth-optimized algorithm for computing set-maximal matches between a database of aligned genetic sequences and the DNA of an individual while respecting the privacy of both the database owner and the individual. We then implement and evaluate our protocol.

**Conclusions:**

Using modern cryptographic techniques, difficult genomic computations are performed in a privacy-preserving way. We enrich this research area by proposing a privacy-preserving protocol for set-maximal matches.

## Background

The abundance of human genomic data in recent years paves the path towards answering very important questions for human nature, such as identifying the genomes responsible for particular illnesses. Simultaneously, the extremely sensitive nature of this data imposes strict restrictions on its use. Fortunately, there is a variety of cryptographic techniques that allow us to create useful yet privacy-preserving systems for computation in genomic data (see [1] for a summary of various techniques). Even though theoretically it is possible to perform every computation in a private way, the generic techniques do not necessarily preserve the efficiency and the accuracy of the original algorithm. Thus, constructing practical privacy-preserving protocols has become a very active area of research.

Quantifying the similarity of genomic sequences is a fundamental problem in genome informatics and there exists numerous proposals for tackling this problem. In this work, we focus on privately computing *set-maximal matches* on genomic data as a way to identify similarity. Initially, research on genomic matching focused on finding efficient and accurate algorithms (e.g. [2–9]). More recently, the issue of privacy has emerged and hence new approaches have been proposed. Freedman et. al. [10] consider the problem of secure keyword-search in a database by relying on a connection to oblivious evaluation of pseudorandom functions. Various other works focus on problems more specific to genomic data. For instance, Jha et al. [11] develop a protocol for securely computing edit distance of DNA sequences, Blanton et al. [12] propose a protocol for outsourcing DNA search via finite automata to multiple computational servers. Baldi et al. [13] focus on similar applications, such as Paternity Testing, and use techniques on private set operations. He et al. [14] construct a protocol that identifies whether two individuals are relatives without revealing any other information about their genomes. More closely related to our work, Shimizu et al. [15] propose a protocol for privately computing set-maximal matches between a database and an individual starting from a genomic position known only to the individual. Even though we also focus on computing set-maximal matches, our problem is more general since our schemes outputs all set-maximal matches without leaking their locations for the client.

The efforts to construct secure but yet efficient and practical protocols for genome analysis have also been reinforced by the establishment of the Integrating Data for Analysis, Anonymization and SHaring (iDASH) privacy and security workshop [16]. Each year, the workshop poses a set of tasks to evaluate the employment of cryptographic techniques for real-world challenges in genome analysis.

In this work, we propose a novel protocol for measuring similarity between a database of DNA sequences and a query DNA sequence privately by identifying the set-maximal matches between the database and the query. This problem was also the competition task on the secure multiparty computation track in the iDASH workshop for 2018 [16].

## Methods

Our goal is to design a protocol for computing the set-maximal matches between a database and a query. We assume that all variable sites are bi-allelic; namely each site has a value in {0,1}. Before reviewing the main cryptographic tools used in this work, we explain our notation and give the formal definition of set-maximal matches.

**Notation** If **M** is a matrix, then **M**_*j*,*i*_ denotes the element of the *j*-th row and *i*-th column. We denote by a bold lowercase letter (e.g. **x**,**y**) a sequence of bi-allelic sites; the *i*-th site of **x** is denoted by **x**[*i*]. Namely, $\mathbf {x} = \left (\mathbf {x}[1],\mathbf {x}[2],\dots, \mathbf {x}[n] \right)$. For simplicity, we use the notation **x**[*i*_1_,*i*_2_] to denote the substring $(\mathbf {x}[i_{1}], \dots, \mathbf {x}[i_{2}])$. A bold uppercase letter (e.g. *yY*) represents a database of genomic sequences; the *j*-th tuple of the database **Y** (i.e. the genomic data of the *j*-th individual in the database) is denoted using a bold lowercase letter as **y**_*j*_. We call *n* the number of sites in our genomic sequences and *m* the number of sequences in the database.

We use the Big *O* notation for describing the limiting behavior of the running time of an algorithm. We say that *f*(*x*)=*O*(*g*(*x*)) if and only if there exists a constant *c* such that for large enough *x*, *f*(*x*)≤*cg*(*x*).

**Definition** Let **Y** be a database and **x** be a query sequence, then a substring **x**[*i*_1_,*i*_2_] with *i*_1_≤*i*_2_ is a set-maximal match between **x** and **Y** if there exists a *j* such that:

1. **x**[*i*_1_,*i*_2_]=**y**_*j*_[*i*_1_,*i*_2_]
2. **x**[*i*_1_−1]≠**y**_*j*_[*i*_1_−1] and **x**[*i*_2_+1]≠**y**_*j*_[*i*_2_+1]

and for any *j*^′^≠*j*, there exists no interval [*i*1′,*i*2′] such that **x**[*i*1′,*i*2′]=**y**_*j*_[*i*1′,*i*2′] and [*i*_1_,*i*_2_] is a strict subset of [*i*1′,*i*2′].

The first two properties assure that the match is a *locally maximal match*, namely that the match cannot be extended in either side and the last property is satisfied when the match is not strictly contained in another match with a different database entry. Sometimes, it is useful to keep only long enough matches; in this case, the definition has an extra parameter called *threshold*.

**Definition** A match is a set-maximal match between **x** and **Y** with threshold *t* if it is a set-maximal match between **x** and **Y** and additionally its length is more than or equal to *t*.

### Cryptographic tools

Secure computation allows multiple parties to compute a joint function while preserving the privacy of their individual inputs. There are feasibility results [17, 18] that allow us to compile any computation into a secure one. Unfortunately, these methods do not preserve the efficiency of the original algorithm. Hence, it is often essential to design novel algorithms that take into account the special nature of secure computation protocols.

**Security model.** Let *P*_0_ and *P*_1_ be two parties holding inputs *x*_1_ and *x*_2_ respectively. They are interested in jointly computing a function *f*(*x*_1_,*x*_2_). A protocol between the two parties A and B, is called secure (or privacy-preserving) if it leaks nothing about the inputs *x*_1_ (to *P*_1_) and *x*_2_ (to *P*_0_) apart from the output value *f*(*x*_1_,*x*_2_) (and the length of the inputs). There are two well-studied adversarial models, the semi-honest and the malicious. A semi-honest adversary observes all communication between the computing parties (and tries to learn information about the inputs), but is not allowed to deviate from the protocol. On the contrary, a malicious adversary is allowed to arbitrarily deviate from the protocol in order to try to learn extra information about the inputs. In both case, the adversary is assumed to be computationally bounded. In this work, the two involved parties *P*_0_ and *P*_1_ are the client and the server and our protocol is secure in the semi-honest model.

In this work, we focus on the Goldreich-Micali-Wigderson (GMW) method [17] for secure computation with *boolean secret shares*. This method gives a way to privately compute XOR (⊕), AND (∧) and NOT (¬) gates in the semi-honest model. Since all computations can be expressed by a circuit containing only these three types of gates, boolean sharing allows to perform every computation in a private fashion. We briefly describe how operations are performed in boolean sharing and some of the available optimizations and implementations.

Intuitively, secret sharing of a value *x* is a split of *x* in many parts such that each part does not reveal any information about *x*, but the knowledge of all the parts allows the recovery of *x*. The GMW framework suggests a specific way to share values such that it is possible to perform any operation on them. Namely, let us assume that there are two parties and each party knows a share of *x*, then using GMW they can end up with shares of any function of *x*.

**Sharing of a bit*b*.** The boolean shares of a bit *b* are two bits 〈*b*〉_0_ and 〈*b*〉_1_ such that 〈*b*〉_0_⊕〈*b*〉_1_=*b*.

**Reconstruction of a bit*b*.** If parties *P*_0_ and *P*_1_ have the shares 〈*b*〉_0_ and 〈*b*〉_1_ respectively, then they reconstruct the bit *b* by exchanging shares and computing 〈*b*〉_0_⊕〈*b*〉_1_.

**Computing XOR privately.** Assume that party *P*_*i*_ knows the secret shares 〈*x*〉_*i*_ and 〈*y*〉_*i*_ of the bits *x* and *y* respectively. Then, party *P*_*i*_ computes a share of *x*⊕*y* by locally computing 〈*x*〉_*i*_⊕〈*y*〉_*i*_.

**Computing AND privately.** Assume that party *P*_*i*_ knows the secret shares 〈*x*〉_*i*_ and 〈*y*〉_*i*_ of the bits *x* and *y* respectively. The shares of *x*∧*y* are evaluated using a *precomputed multiplication triple* (〈*a*〉_*i*_,〈*b*〉_*i*_,〈*c*〉_*i*_) of the bits *a*,*b*,*c* such that *a*∧*b*=*c*. Initially, *P*_*i*_ computes the shares 〈*e*〉_*i*_=〈*a*〉_*i*_⊕〈*x*〉_*i*_ and 〈*f*〉_*i*_=〈*b*〉_*i*_⊕〈*y*〉_*i*_. Then, both parties *P*_0_ and *P*_1_ reconstruct the values *e* and *f*. Finally, *P*_*i*_’s new share is equal to (*i*∧*e*∧*f*)⊕(*f*∧〈*a*〉_*i*_)⊕(*e*∧〈*b*〉_*i*_)⊕〈*c*〉_*i*_.

**Computing NOT privately.** Assume that party *P*_*i*_ knows the secret share 〈*x*〉_*i*_ of the bits *x*. Then, party *P*_*i*_ computes a share of ¬*x* by locally computing 〈*x*〉_*i*_⊕*i*.

We remark that the computation of the multiplication triples does not depend on the actual computation or input, so it can be done in advance during a precomputation phase which requires interaction between the parties. The multiplication triples can be computed using the cryptographic primitive of *random oblivious transfer* [19]. Additionally, we note that computing XOR and NOT gates is done locally and does not require any interaction. On the other hand, an AND operation requires one flow of interaction in order to reconstruct the values *e* and *f*. Therefore, since we can compute many AND operations in parallel, the number of rounds required for computing a function *f* is proportional to the AND-depth of its circuit representation.

Since the introduction of GMW, various optimizations have been proposed [19–21]. Our implementation is based on the ABY framework [22], which provides semi-honest security. Apart from GMW on boolean shares, this framework provides implementations of two other well-studied methods for secure computation, secure computation using arithmetic shares and Yao’s Garbled Circuits. The ABY framework is suitable for mixed-protocols, since it allows for very efficient conversion between the different secure computation methods. Even though our solution is not a mixed-protocol, we use ABY since it includes all known optimizations of GMW and it allows the composition of our protocol with others that are potentially more efficient if implemented using another method of secure computation.

**Two-party secure protocols using the GMW framework.** If party *P*_0_ has input *x*_0_ and party *P*_1_ has input *x*_1_, then they can privately compute a function *f*, which is given in the form of a circuit containing XOR,AND and NOT gates, as follows:

1. Party *P*_*i*_ secret shares *x*_*i*_ by sending a uniformly random binary string *r*_*i*_ with length equal to its input to *P*_1−*i*_ and setting its shares equal to *x*_*i*_⊕*r*_*i*_, where ⊕ denotes the bitwise XOR operation.
2. The two parties compute the function *f* gate-by-gate as described above and end up with boolean shares of the output.
3. The two parties exchange shares and reconstruct the output of the function by computing the XOR of their shares with the shares received by the other party.

By slightly modifying the above protocol, it is possible to achieve *selective reconstruction* of the output, in which case only one of the parties learns the output. For instance, if only the client should learn the output, then at the third step the server sends its shares and the client sends nothing. In this case, the client has enough information to recover the output, whereas the server does not learn the output.

## Results

The GMW framework allows us to transform any computation in a form of a circuit into a privacy-preserving one against semi-honest adversaries. Therefore, we focus on designing a depth-optimized circuit with XORAND and NOT gates to compute set-maximal matches. Then, using the generic protocol described in the previous section, we have a secure protocol for set-maximal matches that requires at most as many rounds of interaction as the depth of the circuit.

### Problem definition

We give an efficient and depth-optimized algorithm for computing set-maximal matches. More specifically, the problem specification is as follows:

**Input:** A genomic database **Y** containing *m* sequences, each of size *n*, a query genomic sequence **x** of size *n*, and a threshold value *t*. **Output:** A matrix **M** of size *m*×*n* such that the element **M**_*j*,*i*_ is equal to the length of the match between **x** and **y**_*j*_ ending at position *i* if the match is set-maximal with threshold *t* and 0 otherwise.

We note that the output as described above leaks the position of set-maximal matches. Because of the very sensitive nature of genomic sequences, it is beneficial to hide even this information. Therefore, we slightly modify the above problem so that the list of set-maximal matches is revealed after applying a random permutation to each row of the output.

**Input:** A genomic database **Y** containing *m* sequences, each of size *n*, a query genomic sequence **x** of size *n*, a threshold value *t* and *m* permutations $(\pi _{k})_{k \in \{1,\dots, m\}}$. **Output:** Let **M** be a matrix of size *m*×*n* such that the element **M**_*j*,*i*_ is equal to the length of the match between **x** and **y**_*j*_ ending at position *i* if the match is set-maximal with threshold *t* and 0 otherwise. The output is the permutation of each row *k* of **M** according to *π*_*k*_.

We observe that the output still reveals the lengths of set-maximal matches for each index. This information could be hidden by applying a random permutation on all the entries of **M**, instead of each row. However, it seems that this would reduce the applicability of the computation, since this information seems central for certain application.

In the secure protocol, the database **Y** and the permutations $(\pi _{k})_{k \in \{1,\dots, m\}}$ are the input of the server, the query **x** is the input of the client and the threshold *t* is known to both parties. To avoid leakage to the server, the protocol has selective reconstruction to the client.

### Algorithm description

We describe our algorithm. We include a sample execution in Fig. 1.

1. We compute a matrix **M** of size *m*×*n* such that **M**_*j*,*i*_ is equal to the length of the match between the query **x** and the database entry **y**_*j*_ ending at position *i* (Fig. 1b).

**Fig. 1:**
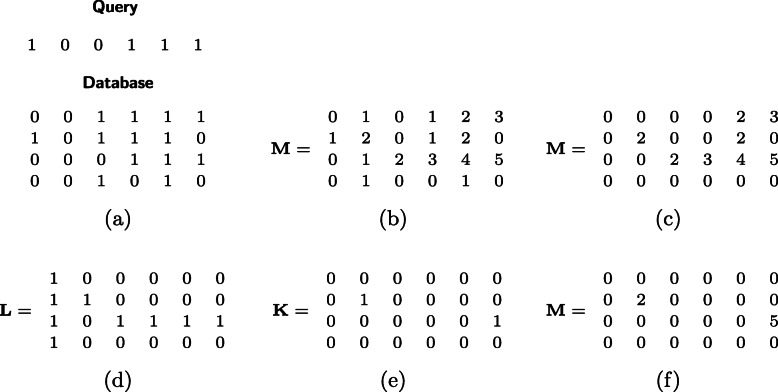
Example execution with threshold *t*=2
2. We set **M**_*j*,*i*_ to 0 if the match of **x** and **y**_*j*_ ending at position *i* is below the threshold *t* (Fig. 1c).
3. We compute a matrix **L** of size *m*×*n* such that **L**_*j*,*i*_ is 0 if there is a *j*^′^≠*j* such that $\phantom {\dot {i}\!}\mathbf {M}_{j',i} > \mathbf {M}_{j,i}$ and 1 otherwise (Fig. 1d).
4. We compute **K** such that **K**_*j*,*i*_ is 0 if there is a *j*^′^ (may be equal *j*) such that $\phantom {\dot {i}\!}\mathbf {L}_{j',i}= y{L}_{j',i+1} = 1$. Namely, there exists a match that is extended to position *i*+1 (Fig. 1e).
5. We set **M**_*j*,*i*_←**M**_*j*,*i*_**K**_*j*,*i*_**L**_*j*,*i*_ and we permute the row **M**_*k*_ according to permutation *π*_*k*_ (Fig. 1f).

After the steps described above, the matrix **M** contains the correct output:

- The output **M** contains the correct length of matches computed in step 1.
- The output **M** contains no matches of length less than *t*, since all such matching have been removed in step 2.
- The output **M** does not output matches strictly contained in another match. If a match is contained in another larger match, then either there is a match with a preceding starting point or a match with a succeeding ending point or both. In the first and third case, this match is excluded in step 3, since there is another database entry with larger value in the corresponding positions of **M**. In the second case, the match is excluded in step 4, since there is another extendable match in the corresponding positions.
- The output **M** contains only locally maximal matches. After step 1, **M** contains the length of matches from their starting position, so it is not possible for a match to be extendable toward a previous position. Additionally, from step 4, **M** does not contain matches extendable towards the next position.

We now describe how to implement each of these steps using boolean circuits with AND,XOR and NOT gates in a depth-optimized way. Compute the length of matches: First, we compute an auxiliary matrix **B**^(0)^ such that $\mathbf {B}^{(0)}_{j,i} = 1$ if **x**[ *i*]=**y**_*j*_[ *i*]. Namely, $\mathbf {B}^{(0)}_{j,i} = \lnot (\mathbf {x}[\!i] \oplus \mathbf {y}_{j}[\!i])$. We observe that **B**^(0)^ indicates whether a match has length greater than or equal to one. Using **B**^(0)^, we compute whether a match has length greater than or equal to two by setting $\mathbf {B}_{j,i}^{(1)} \leftarrow \mathbf {B}_{j,i}^{(0)} \wedge \mathbf {B}_{j,i-1}^{(0)}$. More generally, if **B**^(*k*)^ indicates whether a match has length more than 2^*k*^ or not, then it can be updated to indicate if the length of a match is more than 2^*k*+1^ by setting $\mathbf {B}_{j,i}^{(k+1)} \leftarrow \mathbf {B}_{j,i}^{(k)} \wedge \mathbf {B}_{j,i-(2^{k}-1)}^{(k)}$.

Concurrently, in each iteration we compute a bound on the length of each match by setting **M**^(0)^=**B**^(0)^ and $\mathbf {M}_{j,i}^{(k+1)} \leftarrow \mathbf {B}_{j,i-(2^{k}-1)}^{(k)} \mathbf {M}_{j,i-(2^{k}-1)}^{(k)} +\mathbf {M}_{j,i}^{(k)} $. We observe that this computation gives the actual length of a match if it is less than or equal to 2^*k*^ and returns the lower bound of 2^*k*^ otherwise. Hence, after ⌈log(*n*)⌉ iterations, we set **M**=**M**^(⌈log(*n*)⌉)^.

The addition is computed using a depth-optimized adder, which has AND-depth proportional to the logarithm of the bit length of the numbers returned [23]. Namely, the AND-depth of the adder is *O*(log(log*n*)). Overall, the AND-depth of the length computation is *O*(log(*n*) log(log*n*)). Remove matches with length below the threshold: Let *t*_*k*_≡*t* (mod 2^*k*^) for $k \in \left \{1, \dots, \lceil \log (n)\rceil \right \}$ and $(b_{\lceil \log (n)\rceil }, \dots, b_{1})$ be the bit decomposition of *t*, where *b*_⌈log(*n*)⌉_ is the most significant bit and *b*_1_ is the least significant bit. Let **T**^(*k*)^ be *m*×*n* matrices that indicate candidate matches; initially $\mathbf {T}_{j,i}^{(0)} = 1$ for all *i* and *j*. Similarly to the length computation, we define the auxiliary matrix **B**^(0)^ that initially indicates whether a database and query position match or not. In each iteration, we update **B**^(*k*)^ as in the length computation; namely, we set $\mathbf {B}_{j,i}^{(k)} \leftarrow \mathbf {B}_{j,i}^{(k-1)} \wedge \mathbf {B}_{j,i-(2^{k} - 1)}^{(k-1)} $. In the *k*-th iteration if *b*_*k*_=1, we update $\mathbf {T}^{(k)}_{j,i} \leftarrow \mathbf {B}_{j,i - (t_{k} - 1)}^{(k)}\mathbf {T}_{j,i}$, otherwise **T**^(*k*)^=**T**^(*k*−1)^. Finally, we set $\mathbf {M}_{j,i} \leftarrow \mathbf {M}_{j,i} \mathbf {T}_{j,i}^{(\lceil \log (n) \rceil)}$.

This computation intuitively splits the *t* positions preceding a specific position *i* into parts of increasing powers of two, then it iteratively checks whether each of these parts is a match. Splitting a number into increasing powers of two is equivalent to computing its bit decomposition. Even though the AND-depth of this step is *O*(log(*n*)), it can be performed in parallel to the length computation where we also use the same auxiliary matrix **B**. So, this step does not increase the depth of the circuit. Remove matches contained in other matches: We first compute the maximum length of a match for each position *i* across all the database entries and then perform an equality check between **M**_*j*,*i*_ and the maximum for position *i* to compute each **L**_*j*,*i*_. Each such maximum is computed using the D&C comparison circuit [24] in AND-depth *O*(log(log*n*) log(*m*)). The equality check can be implemented with a depth-optimized circuit in *O*(log(log*n*) log*m*) depth in which the comparison of the bits across the database entries is performed in parallel. Remove extendable matches: A match between **x** and **y**_*j*_ is extendable at position *i* if **B**_*j*,*i*+1_=1. So, in order to remove the extendable matches, we compute $\phantom {\dot {i}\!}\mathbf {K}_{j,i}= \lnot \max _{j'}\left \{\mathbf {L}_{j',i} \wedge \mathbf {L}_{j',i+1}\right \}$ that indicates whether an extendable match exists. Finally, we update **M**_*j*,*i*_←**K**_*j*,*i*_∧**L**_*j*,*i*_∧**M**_*j*,*i*_. Using the D&C comparison, this operation requires *O*(log(log*n*) log*m*)AND-depth. Permute matches: Finally, to remove the information regarding the position of matches, we permute each row *k* of the matrix **M** according to a permutation *π*_*k*_, which is given as an input. All permutations are performed in parallel and require AND-depth *O*(log(*n*)) using the Waksman permutation network [25].

The total AND-depth of the above circuit is *O*(log(*n*)+ log(log*n*) log(*m*)). Since in practice *n*>>*m*, the depth can be assumed to be proportional to *O*(log(*n*)).

We now present an optimization that reduces the output size and the computational cost of the permutations. Even though this does not offer an asymptotic optimization, it is definitely helpful in the experimental efficiency of the protocol.

**Output size reduction and efficient permutations:** Before the permutation step, the above circuit outputs a matrix **M** that contains all set-maximal matches with threshold *t*. We note that the threshold *t* guarantees that there exists no set-maximal matches ending at positions with distance less than *t*. In other words, in each row of the matrix **M** there is at most one none zero element for every *t* positions. By combining every *t* columns of **M** into one equal to their XOR, we reduce the size of the output by a factor of *t* without losing the desired information about set-maximal matches.

After the output reduction, we need to permute each row of a matrix of size *m*×*n*/*t*. Therefore, the Waksman permutation network has depth only *O*(log(*n*/*t*)).

### Experimental evaluation

We have implemented our protocol for secure computation of set-maximal matches using the ABY framework to evaluate its efficiency. We use the network configuration included in ABY for the communication between the two parties and the default method for precomputing multiplication triplets via oblivious transfer. Then, we build the computation circuit gate-by-gate and let ABY handle the sharing procedures and secure computation. The problem has three parameters: *n*, the size of the genomic sequences, *m*, the size of the database and *t*, the threshold of set-maximal matches. We run a series of simulations in a single machine of 16GB RAM and Quad-core 2.8 GHz CPU that simulates both the server and the client to evaluate the efficiency of the protocol with respect to these parameters.

**Fig. 2:**
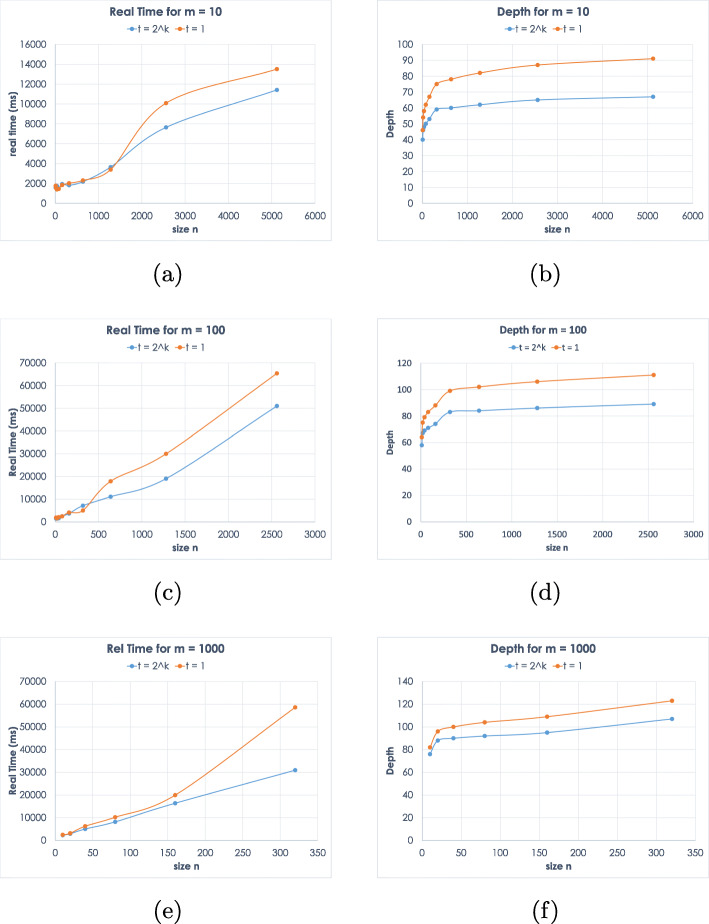
Timings and circuit depths for various values of *n*, *m* and *t*

In our implementation, the server has as input the database, which defines the parameters *n* and *m*, and the threshold value *t*. Similarly, the client’s input is a query of size *n*, the database size *m* and the threshold value *t*. We note that because of the nature of the GMW protocol both parties need to know the values of all three parameters *n*, *m* and *t*. We evaluate how our protocol scales as *n* increases for database size *m*=10,100,1000 and threshold *t*=1 and *t*=2^*k*^, where *k* is the bit length of *n*. Even though the threshold value is an input to the protocol, we plot only two values for clarity of exposition. The two values represent the best and worst values in terms of efficiency. The threshold value 2^*k*^ maximizes the efficiency gain form the output reduction optimization. On the contrary, when the threshold value *t*=1, this optimization is not in use, and hence this value corresponds to the worst case running time. In Fig. 2, we observe that for larger enough values of *n* indeed there is an improvement of both the running time and the depth due to the output reduction technique.

Figure 2a, c, and e show the running time of the protocol for three different values of *m*. For small *n* and *m*, the running time essentially depends on the precomputation phase, but when *m* and *n* are large enough the running time for a given *m* depends almost linearly in *n*. We observe that for small values of *n*, there is almost no difference on the running time for the two threshold values. In this case, the output is reduced by a small factor when *t*=2^*k*^, whereas the precomputation time increases, since the computation circuit needs to include the output reduction. Hence, for small values of *n*, there is no improvement in the efficiency from the output reduction optimization.

In Fig. 2b, d, and f, we notice that the depth depends mainly on log(*n*), which is what was expected by the analysis in the previous section.

## Discussion

Because of the developments on efficiently acquiring DNA data, computing on genomic data has gained a lot of attention in the recent years. At the same time, progress on cryptographic techniques has allowed us to design numerous protocols for private computation that work well in practice. The connection of these two areas of research has led to fascinating directions and applications.

We make progress in an important problem lying in the intersection of these areas concerning the similarity of genomic data. Our motivating application is that of identifying relatives without compromising the privacy of the genomic data of the individuals involved.

## Conclusions

We construct an efficient algorithm for computing set-maximal matches, which is compatible with secure computation approaches. More specifically, our algorithm is designed carefully so that it remains efficient when compiled in the GMW framework, which offers a generic way to perform secure computation in the semi-honest model of security.

We implement and evaluate our algorithm using the ABY framework. Our algorithm runs for relatively large datasets and the behavior of the running time and the rounds of interaction is compatible with our theoretical analysis. The ABY framework is a very general framework that offers many capabilities. Unfortunately, this generality hurts the efficiency of our protocol, so it would be beneficial for the practical efficiency of our scheme to implement it using a tailored secure computation protocol, which is more lightweight and contains only the parts necessary for our protocol.

This work extends an exciting line of research that combines cryptographic techniques for secure computation with efficient and accurate algorithms for genomic analysis. Our main contribution is on securely finding set-maximal matches between a database and a query sequence.

## Data Availability

The code used during the current study is available from the corresponding author on reasonable request.
